# *In silico* analysis of candidate genes associated with humoral innate immune response in chicken

**DOI:** 10.1186/1753-6561-5-S4-S36

**Published:** 2011-06-03

**Authors:** Anna Slawinska, Andrzej Witkowski, Marek Bednarczyk, Maria Siwek

**Affiliations:** 1 Department of Animal Biotechnology, University of Technology and Life Sciences, Bydgoszcz, 85-084, Poland; 2 Department of Biological Bases of Animal Production, University of Life Sciences, Lublin 20-950, Poland

## Abstract

**Background:**

Production and function of natural antibodies (NAbs) constitutes an important mechanism of the humoral innate immunity in vertebrates. The level of NAbs in chicken is heritable and the genetic background has been partly investigated. However, to date the genetic determination of humoral innate immune response in avian species has not been fully described. The goal of this study was to propose a new set of candidate genes with a potential effect on the NAb phenotype for further SNP association study.

**Methods:**

*In silico* analysis of positional and functional candidate genes covered 14 QTL regions associated with LPS, LTA & KLH NAbs and located on six chromosomes: GGA5, GGA6, GGA9, GGA14, GGA18 and GGAZ. The function of the genes was subsequently determined based on the NCBI, KEGG, Gene Ontology and InnateDB databases.

**Results:**

As a result, the core panel of 38 genes participating in metabolic pathways of innate immune response was proposed. Most of them were assigned to chromosomes: GGA14, GGA5, GGA6 and GGAZ (13, 9, 8 and 5 genes, respectively). These candidate genes encode proteins predicted to play a role in (i) proliferation, differentiation and function of B lymphocytes; (ii) TLR signalling pathway, and (iii) MAP signalling cascade.

**Conclusions:**

Proposed set of candidate genes is recommended to be included in the follow-up studies to model genetic networks of innate humoral immune response in chicken.

## Background

Humoral innate immunity in vertebrates that establishes the first barrier against pathogens consists of two basic mechanisms – natural antibodies (NAbs) and complement system. Expanding the knowledge on this field of avian immunology might be of help to overcome the difficulties in poultry industry, struggling constantly with diseases outbreaks eg. Avian Influenza [1]. In chicken, the level of NAbs proved to be heritable [2]. However, the genetic determination of NAbs is not fully described as it lacks information on which genes can be considered as the regulators in the complicated network of NAbs creation and function. This study contributes to the discovery of genetic determination of humoral innate immunity as it lists the proposed positional and functional candidate genes that have the putative impact on the NAb phenotype.

## Methods

Chromosomal regions for *in silico* candidate gene analysis were initially selected based on the location of the QTL associated with the NAb titres directed against LPS (lipopolysaccharide), LTA (lipoteichoic acid) and KLH (keyhole limpet hemocyanine) antigens in chicken. This step was performed based on results from two independent studies, i.e.

• **Study 1** – LPS and LTA NAb QTL detection study [3];

• **Study 2** – LPS and LTA NAb QTL validation study; KLH NAb detection study (data not published).

Study 2 was carried out within a new chicken reference population, set-up as a F_2_ cross between commercially selected breed (WL, White Leghorn) and a Polish, unselected native chicken breed (GP, Green-legged Partridgelike). For a candidate gene analysis reported here, the chromosomal regions of interest included QTL associated with LPS and LTA NAb titres that had been detected in **study 1** and consecutively validated in **study 2** as well as QTL associated with KLH NAb titres that had been detected in **study 2**. These QTL were located in the following chicken chromosomes: GGA5, GGA6, GGA9, GGA14, GGA18 and GGAZ. The regions of interest were designated based on the physical location of the microsatellite markers flanking the QTLs. The list of candidate genes within the QTL regions was prepared based on NCBI database [4], and gene function was assessed with KEGG [5], InnateDB [6] and Gene Ontology [7]. The genes meeting both the criteria, i.e. location within the QTL regions & function in innate immunity (including signalling pathways and B cell function) were listed in a panel of the candidate genes associated with humoral innate immune response.

## Results

The results of the candidate gene analysis are presented in Table 1. Briefly, based on previously described criteria, the total number of 38 candidate genes located on six chromosomes was selected. The highest number of the candidate genes (13 genes) was located on GGA14; 9 genes were found on GGA5 and 8 – on GGA6. Lower number of candidate genes were found on GGAZ (5 genes), on GGA18 (2 genes) and on the GGA9 (1 gene).

**Table 1:** Positional and functional candidate genes associated with innate humoral immune response

Symbol	ID	Name	Ch	Metabolic Pathway	Gene Function
**BLNK**	395733	B cell linker	6	BCR	B-cell development
**CARD11**	416476	caspase recruitment domain family, member 11	14	BCR, TCR, NFκB	NFκB activation
**CASP7**	423901	caspase 7, apoptosis-related cysteine peptidase	6	BCR, TNFα	Apoptosis
**CAT**	423600	Catalase	5	NFκB	Regulation of NFκB activity
**CD59**	423148	CD59 molecule, complement regulatory protein	5	T cells	T cell activation, complement system inhibition
**CD7**	417346	T-cell antigen CD7 precursor	18	T cells	T cell activation, T and B cell interaction, component of mature T cells
**CD82**	423172	CD82 molecule	5	NFκB, p53	Binding of proteins in cell membrane
**CIITA**	427676	class II, major histcompability complex, transactivator	14	TLR, MHC	LRR binding, MHCII transcription activation
**CXCL12**	395180	chemokine (C-X-C motif) ligand 12	6	IL	Leukocyte activation, T cell proliferation, chemotaxis
**FADD**	423146	FAS (TNFRSF6)-associated via death domain	5	NFκB	Apoptosis, NFκB cascade activation, early development of T cells
**FAS**	395274	TNF receptor superfamily, member 6	6	TNFα, Fas, B and T cells	Ig production, immune response with (B cells) Homeostasis between B I T cells
**FGF10**	395432	fibroblast growth factor 10	Z	NFκB, MAPK	TLR activation, inflammatory cytokine secretion (with APC)
**FGF8**	396313	fibroblast growth factor 8	6	MAPK	MAPK cascade activation
**FOS**	396512	v-fos FBJ murine osteosarcoma viral oncogene homolog	5	TLR, BCR, TCR, MAPK, JNK, IL	Synthesis of AP-1 transcription factor
**IGSF6**	771906	immunoglobulin superfamily, mem. 6	14	B and T cells	Membrane receptor of T and B cells
**IL20RB**	768437	interleukin 20 receptor beta	14	Jak-STAT, IL	T and B cells proliferation and differentiation
**IL21R**	416586	interleukin 21 receptor	14	Jak-STAT, IL	T and B cells proliferation and differentiation
**IL31RA**	427140	interleukin 31 receptor A	Z	MAPK, Jak-STAT, IL	MAPKKK cascade, cytokine and chemokine signal transduction, monocyte and macrophage differentiation
**IL4R**	416585	interleukin 4 receptor	14	T cells, IL	Th2 lymhocyte differentation, cytokine receptor
**IL6ST**	395684	interleukin 6 signal transducer	Z	IL	Fragment of cytokine receptor complex
**IL9R**	416587	interleukin 9 receptor	14	Jak-STAT, IL	Jak and STAT activation, cytokine receptor
**JAK2**	374199	Janus kinase 2	Z	Jak-STAT, IL	Cytokine signalling
**LITAF**	374125	lipopolysaccharide induced TNF factor	14	TNFα	TNFα expression
**MAP2K3**	416496	Mitogen activated protein kinase kinase 3	14	MAPK, TLR, JNK, Fc, p38, TNFα, Jak-STAT, TRAIL	MAPKKK cascade
**MAP2K4**	417312	Mitogen activated protein kinase kinase 4	18	MAPK, TLR, Fas, JNK, Fc, TCR, Jak-STAT, TRAIL	MAP kinase activation, in response to different stimuli, survival signal for T cells
**MAP3K1**	427144	mitogen activated protein kinase kinase kinase 1	Z	MAPK, TLR, Fas, JNK, Fc, p38, NFκB, TCR, BCR, INFγ, TRAIL, TNFα	Integration of enzyme fosforylation in response to different factors
**MAP3K 13**	424876	mitogen-activated protein kinase kinase kinase 13	9	MAPK, JNK	Activation of different MAP kinases
**MAPK8 IP3**	426986	mitogen-activated protein kinase 8 interacting protein 3	14	MAPK, JNK	MAPK and JNK integration
**NFKBIA**	396093	nuclear factor of kappa light polypeptide gene enhancer in B-cells inhibitor, alpha	5	TLR, BCR, TCR, NFκB	NFκB Inhibitor
**PDCD4**	374191	programmed cell death 4 (neoplastic transformation inhibitor)	6	JNK	Negative JNK regulation, expression of the gene under control of T cells
**RAG2**	423165	recombination activating gene 2	5	B and T cells	B and T cells differentiation, gene conversion in Ig
**RBP4**	396166	retinol binding protein 4, plasma	6	B cells	Activation of Ig secretion
**SOCS1**	416630	supressor of cytokine sygnalling 1	14	Jak-STAT, IL	Inhibition of cytokine secretion & Jak-STAT cascade
**TCF7L2**	395508	Transcription factor 7-like 2	6	WNT	WNT signalling
**TGFB3**	396438	transforming growth factor, beta 3	5	MAPK, TGFβ, GPCR	MAPK activation, growth factor activity
**TNFRSF13B**	770275	TNF receptor superfamily, member 13B	14	AP-1, NFκB, TNF	Key role in humoral immune response
**TRAF6**	423163	TNF receptor-associated factor 6	5	TNF, TLR, IL, NFκB, TCR	Signal transduction in many pathways, Th1 immune response, T cell activation
**TRAF7**	416555	TNF receptor-associated factor 7	14	TNF	MAPKKK cascade activation

Gene symbol, ID and name according to NCBI database; Ch - chromosome number, Metabolic Pathway and Gene Function based on GO and InnateDB.

It can be summarized that these candidate genes encode proteins predicted to play a role in:

(i) Proliferation, differentiation and function of B lymphocytes, e.g. *CXCL12*, *BLNK*, *IL21R*, *RBP4*, *CD59*, *TNFRSF13B*;

(ii) TLR signalling pathway, e.g. *TRAF6*, *FADD*, *NFκBIA*, *CARD11*, *FAS*, *FGF8*, *TGFB*, *IL31RA*;

(iii) MAP signalling cascade, e.g. *MAP2K3*, *MAP2K4*, *MAP3K1*, *MAP3K13*, *MAPK8IP3*.

## Discussion

Immune response is a complicated process; encoded by multiple genes organized within the frames of functional networks rather than pathways and regulated by many interactions. However, prior to modelling the most probable genetic network, the information is needed on the genes that can be taken into account and their physiological function.

As mentioned above, the function of the proposed set of candidate genes was associated with three groups of cellular and physiological processes that can hypothetically affect innate humoral immune response in chicken. Briefly, production of antibodies, including NAbs takes place in B cells, stimulated by Th2 cytokines. Therefore, both B and T cells function is a crucial element in antibody release. *CXCL12* gene is responsible for B cells proliferation [8]. *CXCL12*^-/-^ knockout mice produced drastically reduced number of B cells and died during the perinatal period [9]. In turn, *BLNK* gene affects B cell development, which was completely inhibited in *BLNK*^-/-^ knockout mouse [10]. Finally, *IL21R* and *RBP4* genes are responsible for maintenance of mature B cells function. Knocked out mice (both *IL21R*^-/-^ and *RBP4*^-/-^) expressed impaired production of antibodies [11,12].

TLR signalling pathway is triggered when molecular patterns (such as LPS or LTA) are recognized. Some of the proposed candidate genes are involved in TLR pathway, just to mention *TRAF6* and *FADD*, as well as genes affecting *NFκB* expression and function, such as *NFκBIA*, *CARD11*, *TNFRSF13B* and *FAS*[13-15]. Furthermore, the analysis *in silico* pointed out a number of genes that activate MAPK cascade, a key signalling pathway initiated by TLR, for example *FGF8*, *TGFB3* and *IL31RA*[14]. Additionally, the candidate gene set includes such genes as *MAP2K3*, *MAPK8IP3*, *MAP3K13*, *MAP2K4* and *MAP3K1*, which are the members of MAPK signal transduction pathway [15].

## Conclusions

Chicken immune response is one of the major areas recently studied in life science research related to livestock. So far, different approaches have been applied to dissect the genetic bases of avian health traits. Rapid development of technology supporting high-throughput genomic studies provided an excellent tool for fast and efficient genotyping. Still, the accurate gene selection can pose a problem. Therefore, the additional criteria, like validated QTL regions may be of assistance to list the proper genes that can be further on evaluated and contribute to genetic network modelling of humoral immune response in chicken. For that reason we proposed a panel of candidate genes related to the level of LPS, LTA & KLH NAbs in chicken.

## Competing interests

The authors declare that they have no competing interests.

## Authors' contributions

AS performed the analysis and drafted the manuscript; AW made substantial contributions to acquisition of data; MB participated in the design of the study; MS conceived of the study, participated in its design and coordination and helped to draft the manuscript.
